# Activation of MT1/MT2 to Protect Testes and Leydig Cells against Cisplatin-Induced Oxidative Stress through the SIRT1/Nrf2 Signaling Pathway

**DOI:** 10.3390/cells11101690

**Published:** 2022-05-19

**Authors:** Junqiang Zhang, Yuan Fang, Dongdong Tang, Xingyu Xu, Xiaoqian Zhu, Shusheng Wu, Hui Yu, Huiru Cheng, Ting Luo, Qunshan Shen, Yang Gao, Cong Ma, Yajing Liu, Zhaolian Wei, Xiaoyu Chen, Fangbiao Tao, Xiaojin He, Yunxia Cao

**Affiliations:** 1 Reproductive Medicine Center, Department of Obstetrics and Gynecology, The First Affiliated Hospital of Anhui Medical University, Hefei 230022, China; ahzjq1006@163.com (J.Z.); 15856389914@163.com (D.T.); zhuxiaoq246@163.com (X.Z.); 15256500584@163.com (H.Y.); chenghuiru008@163.com (H.C.); shenqunshan520@163.com (Q.S.); gaoyang0124med@163.com (Y.G.); congm1995@163.com (C.M.); 13385690561@163.com (Y.L.); weizhaolian_1@126.com (Z.W.); fbtao@ahmu.edu.cn (F.T.); 2 NHC Key Laboratory of Study on Abnormal Gametes and Reproductive Tract, Anhui Medical University, Hefei 230032, China; 3 Key Laboratory of Population Health Across Life Cycle, Anhui Medical University, Ministry of Education of the People’s Republic of China, Hefei 230032, China; 4 Department of Blood Transfusion, Anhui NO. 2 Provincial People’s Hospital, Hefei 230041, China; yuanslience@163.com; 5 Department of Gastrointestinal Surgery, The First Affiliated Hospital of Anhui Medical University, Hefei 230022, China; 13966793402@163.com; 6 Department of Medical Oncology, The First Affiliated Hospital of University of Science and Technology of China, Hefei 230031, China; wushusheng129@gmail.com; 7 Department of Obstetrics and Gynecology, Fuyang Hospital of Anhui Medical University, Fuyang 236000, China; 8 Anhui Province Key Laboratory of Reproductive Health and Genetics, Anhui Medical University, Hefei 230032, China; aydlt98@163.com; 9 Biopreservation and Artificial Organs, Anhui Provincial Engineering Research Center, Anhui Medical University, Hefei 230032, China; 10 Department of Histology and Embryology, Anhui Medical University, Hefei 230032, China; cxyayd@163.com

**Keywords:** melatonin, oxidative stress, melatonin receptor, SIRT1, Nrf2, Leydig cell

## Abstract

There is growing concern that chemotherapy drugs can damage Leydig cells and inhibit the production of testosterone. Increasing evidence shows that melatonin benefits the reproductive process. This study mainly explores the protective effect and possible molecular mechanism of melatonin regarding cisplatin-induced oxidative stress in testicular tissue and Leydig cells. We found that there were only Leydig and Sertoli cells in the testes of gastrointestinal tumor patients with azoospermia caused by platinum chemotherapeutic drugs. Melatonin (Mel) receptor 1/melatonin receptor 2 (MT1/MT2) was mainly expressed in human and mouse Leydig cells of the testes. We also observed that the melatonin level in the peripheral blood decreased and oxidative stress occurred in mice treated with cisplatin or gastrointestinal tumor patients treated with platinum-based chemotherapeutic drugs. iTRAQ proteomics showed that SIRT1/Nrf2 signaling and MT1 proteins were downregulated in cisplatin-treated mouse testes. The STRING database predicted that MT1 might be able to regulate the SIRT1/Nrf2 signaling pathway. Melatonin reduced oxidative stress and upregulated SIRT1/Nrf2 signaling in cisplatin-treated mouse testes and Leydig cells. Most importantly, after inhibiting MT1/MT2, melatonin could not upregulate SIRT1/Nrf2 signaling in cisplatin-treated Leydig cells. The MT1/MT2 inhibitor aggravated the cisplatin-induced downregulation of SIRT1/Nrf2 signaling and increased the apoptosis of Leydig cells. We believe that melatonin stimulates SIRT1/Nrf2 signaling by activating MT1/MT2 to prevent the cisplatin-induced apoptosis of Leydig cells.

## 1. Introduction

Chemotherapy is a well-recognized effective treatment for various cancers. However, cancer chemotherapy medications have unavoidable adverse effects, such as reproductive damage and genotoxicity [1]. Chemotherapy has long been linked to infertility [2]. The 5-year clinical follow up of cisplatin-treated testicular cancer patients revealed that the dysfunction of testicular Leydig cells led to an increased risk of long-term testicular failure [3]. Some studies have shown that chemotherapy can not only aggravate infertility in patients with testicular cancer, but also leads to lifelong low fertility/azoospermia in many cancer survivors [4]. Recently, it was observed that chemotherapy greatly reduced the ability of the testis to produce testosterone in animal experiments [5]. Chemotherapy leads to the increased production of reactive oxygen species (ROS), which cause oxidative stress. Clinically, high-dose chemotherapy drugs are often used to treat various types of tumors. Some studies have shown that even small doses of chemotherapeutic drugs damage spermatogenic cells [6,7]. High doses of chemotherapeutic drugs can lead to a significant and persistent decline in the function of normal Leydig cells, reduced androgen production, and irreversible damage to fertility [8,9]. Leydig cells are an essential site for testosterone (T) synthesis. The luteinizing hormone (LH) promotes testosterone synthesis by activating Leydig cells. Testosterone has the effect of promoting spermatogenesis and the development of male reproductive organs [10]. In addition, a randomized controlled trial found that chemotherapy drugs can not only reduce the level of testosterone, but also increase the levels of follicle-stimulating hormone (FSH) and luteinizing hormone in the peripheral blood of patients with rectal cancer [11]. Therefore, it is necessary to prevent the effects of chemotherapeutic drugs on the function of Leydig cells.

Cisplatin (CP) is the most frequently used platinum-based compound that inhibits DNA transcription and replication by inducing DNA crosslinking and DNA double-strand breaks, leading to programmed cell death/apoptosis, thereby exerting its anti-tumor effect [12]. Carboplatin and oxaliplatin can be used as alternatives to cisplatin; they are considered to pose a low risk of ototoxicity [13,14]. Furthermore, CP causes oxidative stress by releasing ROS, which leads to cell death and necrosis [15]. Recently, some studies have reported that CP could significantly increase the levels of malondialdehyde (MDA) and reduce those of glutathione (GSH). It also reduced the activities of superoxide dismutase (SOD), catalase (CAT), and glutathione peroxidase (GSH-Px) in rat testes [16,17,18]. In addition, cisplatin alone or in combination with vinblastine and bleomycin decreased the plasma testosterone levels and increased the FSH and LH levels in rats [19,20,21]. These findings indicate that oxidative stress may play a role in CP-induced reproductive toxicity and genotoxicity. Nevertheless, the specific regulatory mechanism of CP-induced damage to the testes and Leydig cells remains unclear.

Melatonin (Mel) is a hormone secreted by the pineal gland. It regulates the circadian rhythm, oxidative stress, the immune response, and reproduction [22]. Melatonin is produced in many tissues, including the gastrointestinal system, skin, leukocytes, bone marrow, placenta, ovaries, and testes [23]. Furthermore, melatonin inhibits the destruction of normal cells by chemotherapeutic drugs during chemotherapy for reproductive or non-reproductive cancers and amplifies the pathway associated with chemotherapeutic-drug-induced cancer cell death [24,25]. Melatonin also reduces cisplatin-induced testicular and renal damage [26]. In terms of the mechanism, melatonin not only scavenges oxygen free radicals directly through its non-receptor pathway, but it also regulates oxidative stress through antioxidant enzymes mediated by its receptor pathway [27]. Melatonin plays a regulatory role through melatonin receptor type 1 (MT1) and melatonin receptor type 2 (MT2), which are G-protein-coupled membrane-bound receptors [28]. It has been found that the activation of MT1 and MT2 can lead to alterations in the level of second messengers that control intracellular signal transduction [29] and apoptosis [30,31]. MT1 and MT2 can also activate different signal cascades in various tissues, organs, or species [32].

The deacetylase silencing information regulator 1 (SIRT1) is involved in antioxidant, anti-inflammatory, and anti-apoptotic pathways by deacetylating a range of proteins [33]. SIRT1 scavenges ROS mainly by regulating Cu/Zn Superoxide Dismutase (SOD1) and manganese superoxide dismutase (SOD2) [34]. In one study, SIRT1 expression was downregulated in the ovaries of MT1-KO mice [35]. When cells are stimulated by oxidative stress, the nuclear factor erythroid 2-related factor 2 (Nrf2) plays an important role in cell survival [36,37]. Nrf2 is a transcription factor that regulates the expression of its downstream proteins heme oxygenase 1 (HO-1) and NAD(P)H quinone oxidoreductase-1 (NQO1) [38]. HO-1 and NQO1 are important antioxidant defense signals to prevent oxidative stress [37]. Furthermore, Kim et al. hypothesized that melatonin might activate Nrf2 signaling by binding to MT2, leading to catalase overexpression during oocyte maturation [39]. Luzindole, an MT1/MT2 melatonin-receptor inhibitor, decreased the expression of the Nrf2 gene in adipose-derived mesenchymal stem cells [40]. Some studies showed that melatonin can enhance SIRT1 and Nrf2 activity [37,41,42]. SIRT1 and Nrf2 appear to be mainly regulated by melatonin. However, whether melatonin receptors play a role in SIRT1 and Nrf2 regulation in the testes and Leydig cells remains unknown.

The purpose of this study was to determine the effect of platinum chemotherapeutic drugs on gonadotropin toxicity. Meanwhile, the effects of platinum chemotherapeutic drugs on testis and Leydig cells, as well as the expression and localization of melatonin receptors, were studied in gastrointestinal tumor patients with azoospermia caused by platinum chemotherapeutic drugs. Subsequently, we used cisplatin-induced adult male mice as an in vitro model to study the protective effects of melatonin on male mouse testis and gonadal damage. To study whether melatonin protects Leydig cells from cisplatin-induced damage through SIRT1/Nrf2 signaling mediated by melatonin receptors (MT1/MT2).

## 2. Materials and Methods

### 2.1. Chemicals and Reagents

Melatonin (Cat#: 73-31-4) and cisplatin (Cat#: 15663-27-1) were purchased from Sigma-Aldrich (St Louis, MO, USA), whereas the melatonin-receptor antagonist Luzindole (Cat#: 117946-91-5) and melatonin-receptor agonist Ramelteon (Cat#: 196597-26-9) were purchased from MedChemExpress, LLC (Medchemexpress, Shanghai, China). The antibodies used in this study were as follows: anti-melatonin receptor 1 (Cat#: ab203038) and anti-melatonin receptor 2 (Cat#: ab203346) were obtained from Abcam (Cambridge, MA, USA). Anti-ASMT (Cat#: abx005008) was obtained from abbexa (Cambridge, UK). Anti-AANAT (Cat#: 17990-1-AP) was obtained from Proteintech (Chicago, IL, USA). The anti-Nrf2 antibody (Cat#: PB9290) was purchased from BOSTER (Wuhan, China). Anti-Bcl-2 (Cat#: 3498S), anti-Bax (Cat#: 2772S), and anti-β-Actin (Cat#: 4970S) were obtained from Cell Signaling Technology (Beverley, MA, USA). Anti-HO-1 (Cat#: AF5393) and anti-NQO1 (Cat#: DF6437) were obtained from Affinity (Affinity Biosciences, Pottstown, OH, USA). Anti-3β-HSD (Cat#: sc-515120), Anti-SOD1 (Cat#: sc-101523), and Anti-SOD2 (Cat#: sc-137254) were obtained from Santa Cruz (Dallas, CA, USA). A Mouse MT (melatonin) ELISA Kit (Cat#: E-EL-M0788c), Human MT (melatonin) ELISA Kit (Cat#: E-EL-H2016c), GnRH (gonadotropin-releasing hormone) ELISA Kit (Cat#: E-EL-0071c), T (testosterone) ELISA Kit (Cat#: E-EL-0155c), Human FSH (follicle stimulating hormone) ELISA Kit (Cat#: E-EL-H1143c), and Human LH (luteinizing hormone) ELISA Kit (Cat#: E-EL-H6019) were purchased from Elabscience (Wuhan, China). A Lipid Peroxidation MDA Assay Kit (Cat#: S0131S), Total Superoxide dismutase Assay Kit (Cat#: S0101S), and Total Antioxidant Capacity Assay Kit (Cat#: S0121) were obtained from Beyotime (Shanghai, China).

### 2.2. Study Participants

In the study, 30 healthy Chinese male volunteers participated as part of the control group. The mean age was 56.1 ± 7.46 years (range: 44–68 years). In addition, 30 male patients diagnosed with gastrointestinal tumors for the first time were categorized as the tumor group. The mean age was 55.7 ± 6.44 years (range: 43–67 years). Finally, 30 male gastrointestinal tumor patients treated with chemotherapy drugs (cisplatin or oxaliplatin) at least twice were categorized as the tumor + CT group. The mean age was 56.6 ± 8.79 years (range: 47–68 years). The gastrointestinal tumor patients included those with gastric cancer and colorectal cancer. The inclusion criteria were as follows: (1) gastric cancer and colorectal cancer had been confirmed according to the 2019 WHO classification of tumors of the digestive system; and (2) all of the patients had been diagnosed and operated on for the first time, and there was no history of anticancer treatment, such as radiotherapy, chemotherapy, targeted therapy, and biotherapy before operation. The exclusion criteria were as follows: (1) patients who also had tumors of other tissues and organs; (2) patients who had underlying diseases, such as hypertension, diabetes, and cardiovascular diseases, combined with severe infection and immune system diseases; (3) patients with incomplete clinical data (routine blood tests, liver and kidney function, five indicators of gastrointestinal tumors, etc.); and (4) patients with hematological diseases, abnormal results from routine blood tests, etc. Three testicular tissue samples of obstructive azoospermia (OA) patients were used as normal spermatogenesis controls, as testicular tissue from normal fertile people could not be obtained in accordance with the rules of the Medical Ethics Committee. Meanwhile, three testicular tissue samples from gastrointestinal tumor patients with azoospermia caused by platinum chemotherapeutic drugs were used as a chemotherapy group. All subjects provided informed consent. The sera of the above volunteers and patients were collected and stored at −80 °C for follow-up experiments.

### 2.3. Animal Experiments and Design

Eighty male C57BL/6J mice, approximately 8 weeks of age and 20–28 g in weight, were obtained from GemPharmatech Co., Ltd. (Nanjing, China). After 1 week of adaptive feeding, the mice were randomly divided into 2 experimental groups and kept under controlled conditions (22 ± 2 °C, 12 h/12 h light/dark cycle).

Experiment 1. To explore whether CP can cause testicular injury and whether CP-treated mice can self-recover. Forty-eight mice were randomly divided into 8 groups (6/group):

- Control d0 group and CP d0 group: The mice were administered an intraperitoneal injection of physiological saline (same volume as for the CP d0 group) and CP (3 mg/kg) for 4 consecutive days [1]. Then, the mice were sacrificed;
- Control d8 group and CP d8 group: The mice were administered an intraperitoneal injection of physiological saline (same volume as for the CP d8 group) and CP (3 mg/kg) for 4 consecutive days. Then, the mice were sacrificed on the 12th day;
- Control d17 group and CP d17 group: The mice were administered an intraperitoneal injection of physiological saline (same volume as for the CP d17 group) and CP (3 mg/kg) for 4 consecutive days. Then, the mice were sacrificed on the 21st day;
- Control d34 group and CP d34 group: The mice were administered an intraperitoneal injection of physiological saline (same volume as for the CP d34 group) and CP (3 mg/kg) for 4 consecutive days. Then, the mice were sacrificed on the 37th day.

Experiment 2. To determine whether melatonin could protect against CP-induced testicular damage. Thirty-two mice were randomly assigned into 4 groups, each with 8 mice: control, CP, CP + Mel, and Mel. Except for the control and CP groups, all of the mice were administered an intraperitoneal injection the of melatonin (10 mg/kg) for 21 days [43]. Except for the control and Mel groups, all groups received an intraperitoneal injection of CP (3 mg/kg) once daily for 4 days, commencing on the first day. The mice that had been given saline as a treatment were used as controls. All animal operations followed the Association of Laboratory Animal Sciences at Anhui Medical University’s criteria for humane care. These studies were approved by the Animal Ethics Committee of Anhui Medical University.

### 2.4. Blood and Tissue Collection

Peripheral blood was taken in a centrifuge tube, adding heparin as an anticoagulant. The blood was centrifuged and the serum was separated and stored at −80 °C. The testes and epididymides were quickly removed from the mice and weighed. After weighing, one testis and epididymis were fixed in 4% paraformaldehyde for histopathological and immunohistochemical (IHC) staining, while the other testis was preserved at −80 °C for immunoblotting and oxidative stress experiments.

### 2.5. Sperm Count

Mouse epididymides were isolated, cut into tissue blocks in 1 mL PBS, and cultured at 37 °C for 5 min to release sperm. After centrifugation to remove the supernatant, the sperm was placed in phosphate-buffered saline for determining the sperm concentration. A hemocytometer grid was used for sperm counts according to the WHO laboratory manual. Each sample count was completed by two technicians.

### 2.6. Protein Extraction, iTRAQ Labeling, and Proteomic Analysis

The samples H-3, H-5, H-7, Dis-2, Dis-5, and Dis-8 were used for protein extraction. The total proteins were extracted by the cold acetone method, and their concentrations were determined using the Bradford method. To a 1.5 mL centrifuge tube, 100 μg of the proteins was added. The proteins were digested using Trypsin Gold (Promega, Madison, WI, USA) at 37 °C for 4 h in the following ratio of protein: trypsin = 20:1. Using 8-plex iTRAQ reagent (Applied Biosystems, Foster City, CA, USA), the peptides were processed according to the manufacturer’s methodology. H-3, H-5, and H-7 were given the tags 113, 114, and 115, respectively, whereas Dis-2, Dis-5, and Dis-8 were given the tags 116, 117, and 118. Using an LC-20AD HPLC pump system (Shimadzu, Kyoto, Japan), the labeled pooled peptides were dried and redissolved for fractionation by strong cation-exchange chromatography. A nanoESI source ionized the peptides separated by liquid-phase chromatography and then delivered them to a tandem mass spectrometer (Orbitrap Exploris 480, Thermo Fisher Scientific, San Jose, CA, USA) for data-dependent acquisition (DDA) mode detection. The MS1 mass spectrometer scanning range was 350 to 1600 *m*/*z*, the resolution was set to 60,000, the starting *m*/*z* of MS2 was fixed at 100, and the resolution was set at 15,000. The MS2 fragmentation ion screening conditions were as follows: charge, 2+ to 7+, and the first- and second-parent ions with a peak intensity greater than 50,000. HCD was used to fragment the ions, and an Orbitrap was used to detect the fragmented ions. The dynamic exclusion time was set to 30 s. The AGC settings were MS1 1E6 and MS2 1E5. The Mascot 2.3.02 search engine was used for protein identification and quantification (Matrix Science, Boston, MA, USA). For protein identification, a mass tolerance of 20 Da (ppm) was allowed for intact peptide masses, and a mass tolerance of 0.05 Da was allowed for fragmented ions; one missed cleavage in the trypsin digests was allowed. The variable modifications included oxidation (M) and iTRAQ8plex (Y), whereas the fixed changes included carbamidomethyl (C), iTRAQ8plex (N-term), and iTRAQ8plex (K). Protein quantification was performed for all unique peptides (at least one unique spectrum). The peptides with isobaric tags were quantitatively analyzed using the automated program IQuant [44]. A mascot percolator was added to enable reliable significance measurements. The peptide-spectrum matches (PSMs) were pre-filtered at a PSM-level false-discovery rate (FDR) of 1% to measure the confidence of the peptides. After protein inference, a protein FDR of 1%, based on the picked protein FDR approach [45], was predicted to limit the number of false positives at the protein level (protein-level FDR ≤ 0.01). The median protein ratio was used to weigh and adjust the quantitative protein ratios in Mascot. Only ratios with fold changes of >1.1 or <0.9 were employed, and Q-values of < 0.05 were considered significant [46]. The Gene Ontology (GO) database (http://en.wikipedia.org/wiki/Gene Ontology, accessed on 31 October 2021) is a global standard for gene functional categorization systems. The pathways were used as search parameters in the KEGG pathway database (http://www.genome.jp/kegg/pathway.html, accessed on 31 October 2021). The heatmap package in R was used to perform the heatmap/hierarchical clustering of differently expressed proteins (DEPs). PPI networks for the overlapping prognostic DEPs were generated by the STRING database (version 11.0).

### 2.7. Isolation and Culture of Testicular Leydig Cells

Testicular Leydig cells were acquired as described by Klinefelter et al. [47] and Chemes [48], with appropriate modifications. The main steps were as follows: aseptically collect mouse testes and remove the capsule and blood vessels on ice. After rinsing using the DMEM/F12 (Gibco, Waltham, MA, USA), gently disperse the testis parenchyma with tweezers and digest with 0.5 g/L type-I collagenase (Solarbio, Beijing, China) at 34 °C for 30 min with shaking. Wash with the culture medium four times to terminate the digestion. Using 200-mesh and 400-mesh screen filters, centrifuge the filtrate at 1500 r/min for 10 min, and discard the supernatant. Dilute the cell pellet with 2 mL of DMEM/F12. Add the cell suspension to four-layer density gradient Percoll (Solarbio, Beijing, China) separation solutions (from the bottom of the separation tube upward: 60%, 37%, 26%, and 21%) at 4 °C. Centrifuge the suspension at 3000 r/min for 30 min. Collect between 37% and 60% of the cells, dilute them using DMEM/F12, centrifuge at 1500 r/min for 10 min, add the pellet to the culture medium containing fetal calf serum, and shake well.

Melatonin was dissolved in cell medium after being diluted in DMSO (1 M) (Macklin, Shanghai, China) to form a stock solution. Cisplatin was dissolved in water to form a stock solution (8 mg/mL). To control for the influence of DMSO, in each in vitro experiment, cell medium containing 0.1% DMSO only was included. The in vitro investigation was separated into three different studies. The goal of this study was to determine if cisplatin inhibited SIRT1/Nrf2 signaling and melatonin production. Leydig cells were stimulated using cisplatin at concentrations of 0, 2, 4, 8, 16, and 32 μg/mL for 24 h. To stimulate cell damage, we chose an 8 μg/mL cisplatin concentration after referring to a previous study [9]. To explore whether melatonin relieved cisplatin-induced cell damage, cells were pretreated using melatonin (0.1, 1, and 10 μM) or ramelteon (10 nM) before cisplatin treatment [49]. To investigate the role of the MT1/MT2 melatonin receptors in cisplatin-induced cell damage, cells were pretreated using Luzindole (10 μM) before cisplatin stimulation [50].

### 2.8. Histological Evaluation of Testes

After the mouse testes and epididymides were fixed with 4% formaldehyde, they were embedded in paraffin. Then, serial sections of 4 μm were cut with a microtome and dyed using hematoxylin and eosin (H&E). To morphologically analyze the testes and epididymides, the dyed sections were examined under a light microscope (Axio Scan Z1, Zeiss, Germany).

### 2.9. ELISA Assay

The levels of gonadotropin-releasing hormone (GnRH), FSH, LH, T, and Mel were detected according to the manufacturer’s instructions. Adult male human serum was collected for the subsequent detection of the GnRH, FSH, LH, T, and Mel levels. The frozen mouse testicular tissue was thawed, and then 100 mg of the tissue was homogenized in 1 mL of PBS. After centrifugation at 12,000 rpm and 4 °C for 10 min, the supernatant was collected for the detection of the contents of testosterone and melatonin.

### 2.10. Biochemical Markers of Oxidative Damage Assay

The activity of SOD and total antioxidant capacity (T-AOC), as well as the content of MDA, in the plasma of male mice and the supernatants of testicular tissue homogenate were measured according to the manufacturer’s instructions. Leydig cells were collected and treated with 10 µM DCFH-DA diluted in serum-free medium at 37 °C for 20 min to determine the ROS. The cells were then washed three times in PBS and resuspended. Flow cytometry was used to detect the fluorescence using the FITC channel with an excitation wavelength of 488 nm. The data analysis was performed in Flowjo software (FlowJo, LLC., Ashland, OR, USA).

### 2.11. TUNEL Staining

The Colorimetric TdT-mediated dUTP Nick-End Labeling (TUNEL) Apoptosis Assay Kit (Cat#: C1098; Beyotime, Shanghai, China) was used to examine cell DNA damage. For sections, the mouse testes were preserved in 4% formaldehyde and embedded in paraffin. The TUNEL Apoptosis Assay Kit was used to color the testicular sections (4 μm) after they were rinsed in distilled water. The testicular slides were permeabilized for 20 min at room temperature with 0.1% Triton X-100. The samples’ endogenous peroxidase was then inactivated for 10 min with 0.3% H_2_O_2_ in PBS. The TUNEL reaction mixture was incubated at 37 °C for 60 min before being stained with streptavidin-HRP at room temperature for 30 min. A hematoxylin staining solution was used to visualize the nuclei. TUNEL-positive cells were dyed brown, while the nuclei were stained blue, and the samples were examined using a light microscope (Axio Scope A1, Zeiss, Germany). The number of brown cells out of the total number of cells was used to calculate the percentage of TUNEL-positive cells.

### 2.12. Immunohistochemistry

To fix testicular portions, 4% paraformaldehyde was used. The dried tissue was then embedded in paraffin. The testes were chopped into 4 μm chunks and placed on glass slides. After performing antigen retrieval and quenching endogenous peroxidases, normal goat serum working solution was added for blocking. Primary antibodies (1:400 dilution) were applied to these slides overnight at 4 °C. The slides were incubated with a secondary antibody on the second day, and then an SP reaction (ZSGB-BIO, Beijing, China), a DAB reaction (ZSGB-BIO, Beijing, China), and hematoxylin restaining were performed.

### 2.13. Immunofluorescence

The Leydig cell coverslips and testicular sections (4 μm) were fixed with 4% (*v/v*) paraformaldehyde for 30 min at 37 °C The slides were blocked using Immunol Staining Blocking Buffer (Beyotime, Shanghai, China) before being incubated for 16–18 h at 4 °C with the primary antibodies anti-MT1/-MT2 (1:500 dilution) and anti-3β-HSD (1:500 dilution). The slides were incubated with secondary antibodies conjugated with Alexa Fluor 488 and Alexa Fluor 594 for 60 min at room temperature after being washed with PBS (1:400 dilution; ZSGB-BIO, Beijing, China). Finally, the nuclei were stained with DAPI (Thermo, 1:5000 dilution) for 5 min at room temperature. Then, the slides washed twice with PBS. The fluorescence images were captured using digital slide-scanning equipment (model: VS200, SLIDEVIEW, Olympus, Tokyo, Japan).

### 2.14. RT-PCR

The total testicular RNA was isolated using Trizol reagent from azoospermia (OA) patients and gastrointestinal tumor patients with azoospermia caused by platinum chemotherapeutic drugs. Genomic DNA was removed using DNase enzymes without RNase. The reverse transcription of mRNA was performed with a PrimeScript™ RT reagent Kit (Takara, Cat#: RR037A). Real-time RT-PCR was implemented with SYBR Green I on a LightCycler 480 instrument. The expression of the target gene mRNA was detected with the level of β-actin mRNA as the normalization. The sequences of human MT1 gene-specific primers were F: 5′-CTGTCGGTGTATCGGAACAAG-3′ and R: 5′-CCAACGGGTACGGATAAATGG-3′. The sequences of human MT2 gene-specific primers were F: 5′-ACGACCCACGCATCTATTCC-3′ and R: 5′-ACGACAGCGATAGGGAGGAG-3′. The sequences of human β-actin gene-specific primers were F: 5′- GATCATTGCTCCTCCTGAG-3′ and R: 5′-CTAAGTCATAGTCCGCCTAG-3′.

### 2.15. Annexin V-FITC Apoptosis Detection

For apoptosis detection, Leydig cells were evaluated using an annexin V-FITC apoptosis detection kit (BD, Cat#: 556547). Leydig cells were digested by trypsin without EDTA. Then, the cells were washed twice with PBS. After centrifugation, the cells were suspended in 500 μL of binging buffer. Subsequently, 10 μL of annexin V-FITC and 5 μL of PI were added to the binging buffer at room temperature for 10 min. Stained cells were detected by flow cytometry (BD FACSVerse, San Jose, CA, USA). The data analysis was performed in Flowjo software (FlowJo, LLC., Ashland, OR, USA).

### 2.16. Immunoblotting

Protein was recovered from mouse testicular and Leydig cells using a RIPA lysis solution, including a phosphatase inhibitor cocktail and the protease inhibitor phylmethanesulfonyl fluoride (PMSF). A BCA protein assay kit (Beyotime, Cat#: P0012) was used to determine the protein concentration. The total protein was denatured by boiling. The protein (30–50 g per well) was separated with 8–15% SDS-PAGE. The protein was electrophoretically separated and then transferred to a PVDF membrane (Millipore, Burlington, VT, USA). The membrane was incubated with the primary anti-Bcl-2 antibody (1:2000 dilution), anti-Bax antibody (1:2000 dilution), anti-Nrf2 antibody (1:1000 dilution), anti-HO-1 antibody (1:1000 dilution), anti-NQO1 antibody (1:1000 dilution), anti-SIRT1 antibody (1:1000 dilution), anti-SOD1 antibody (1:2000 dilution), anti-SOD2 antibody (1:2000 dilution), anti-MT1 antibody (1:2000 dilution), anti-MT2 antibody (1:2000 dilution), anti-ASMT antibody (1:2000 dilution), anti-AANAT antibody (1:2000 dilution), and anti-β-actin antibody (1:1000 dilution) for 16–18 h at 4 °C. Then, the membrane was incubated with an HRP-conjugated secondary antibody (1:5000 dilution; Elabscience, Wuhan, China) raised against distinct primary antibodies after washing in TBST. The ChemiDocTM Touch Imaging System of Bio-Rad was used to image the protein bands (Hercules, CA, USA).

### 2.17. Statistical Analysis

The data were analyzed using the SPSS23.0 statistical software and expressed as the mean ± SEM. The differences between two groups were analyzed using a *t*-test. Three or more groups were compared with a single-factor analysis of variance (one-way ANOVA). Values of *p* < 0.05 and *p* < 0.01 were considered statistically significant.

## 3. Results

### 3.1. Effects of Platinum Chemotherapeutic Drugs on the Levels of Gonadotropin, Melatonin, and Oxidative Stress in the Peripheral Blood of Male Gastrointestinal Tumor Patients, and Reducing the Expression of MT1/MT2 in the Testis

**Figure 1:**
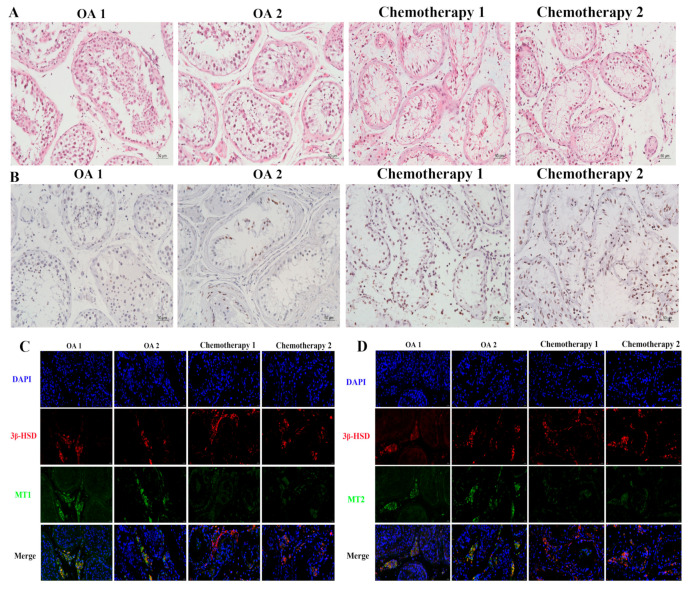

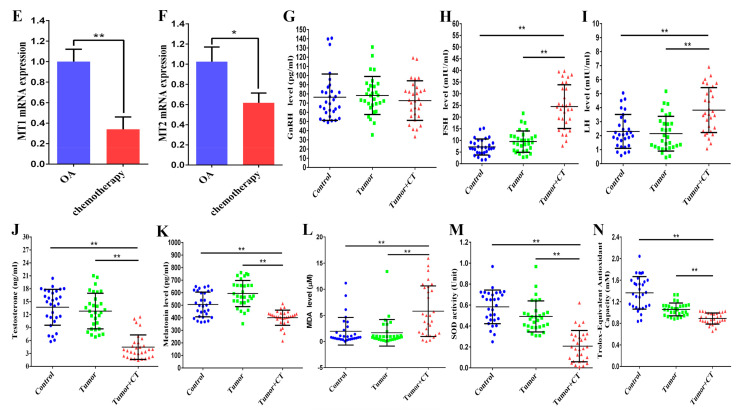
Effects of platinum chemotherapeutic drugs on the levels of gonadotropin, melatonin, and oxidative stress in the peripheral blood of male gastrointestinal tumor patients, and reducing the expression of MT1/MT2 in the testis. (**A**) Human testicular tissue was stained with H&E (scale bar = 50 μm). (**B**) Images of TUNEL staining in human testicular tissue (scale bar = 50 μm). (**C**) Representative immunofluorescent pictures of MT1 in human testicular tissue; 3β-HSD was tagged with red fluorescence and MT1 was tagged with green fluorescence. The nucleus was labeled with DAPI (scale bar = 20 μm). (**D**) Representative immunofluorescent pictures of MT2 in human testicular tissue; 3β-HSD was tagged with red fluorescence and MT2 was tagged with green fluorescence. The nucleus was labeled with DAPI (scale bar = 20 μm). (**E**) MT1 mRNA expression in human testicular tissue. (**F**) MT2 mRNA expression in human testicular tissue. (**G**) GnRH level in human serum. (**H**) FSH level in human serum. (**I**) LH level in human serum. (**J**) Testosterone level in human serum. (**K**) Melatonin level in human serum. (**L**) Content of MDA in human serum. (**M**) SOD activity in human serum. (**N**) Total antioxidant capacity in human serum. Data are presented as the mean ± SEM. *N* = 3 cases per group in (**A**–**F**). *N* = 30 cases per group in (**G**–**N**). CT, chemotherapy. * *p* < 0.05, ** *p* < 0.01 versus the control group or the tumor group.

Human serum (*N* = 90) was divided into three groups for detection: the healthy population group (Control) (*N* = 30), the group of gastrointestinal tumor patients (Tumor) (*N* = 30), and the group of gastrointestinal tumor patients treated with platinum-based chemotherapy drugs (Tumor + CT) (*N* = 30). Compared with the control group, only Leydig and Sertoli cells were observed, and spermatogenic cells were not found in the chemotherapy group (Figure 1A). We also found that the damage to Leydig and Sertoli cells was increased in the chemotherapy group (Figure 1B). The immunofluorescence results showed that MT1 and MT2 were mainly expressed in Leydig cells, and their mRNA expression decreased in the chemotherapy group (Figure 1C–F). The negative control results of MT1 and MT2 immunofluorescence in human testicular tissue are shown in Figure S1A. There were no differences in the levels of oxidative stress markers, melatonin, and gonadal hormones between the control group and the tumor group. Likewise, there were no differences in the GnRH levels among the three groups (Figure 1G). However, the levels of FSH and LH were significantly increased in the Tumor + CT group (Figure 1H,I). Compared to the control group, the levels of testosterone and melatonin were significantly reduced in the tumor + CT group (Figure 1J,K). The level of MDA, a marker of oxidative stress, was increased in the Tumor + CT group. The SOD activity and T-AOC were decreased in the Tumor + CT group (Figure 1L–N). Based on these data, we propose that platinum chemotherapeutic drugs cause testicular injury and decrease the expression of MT1 and MT2 in Leydig cells.

### 3.2. Cisplatin Treatment Resulted in Damage to the Testes of Male Mice and Increased Oxidative Stress

**Figure 2:**
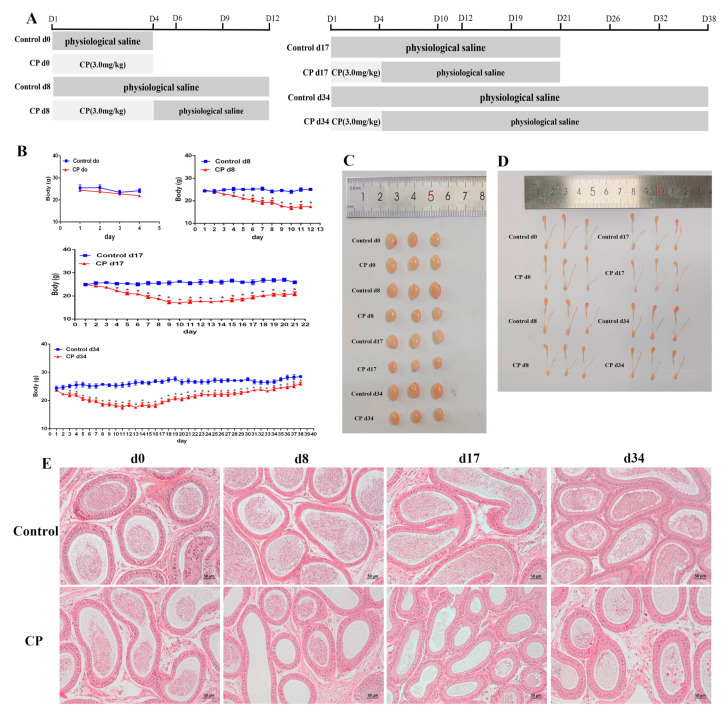

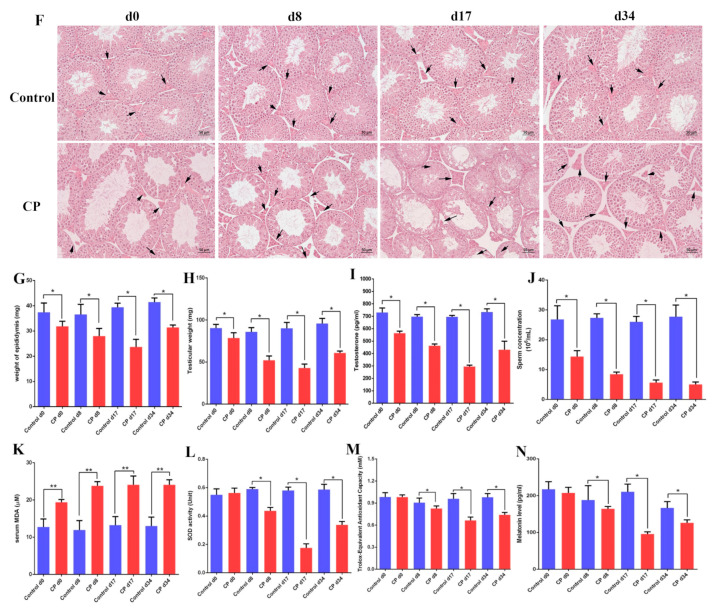
Cisplatin treatment resulted in damage to the testes of male mice and increased oxidative stress. (**A**) Experimental design. (**B**) Changes in the body weight of mice during different recovery periods after cisplatin treatment. (**C**) Images of mouse testes sizes. (**D**) Images of mouse epididymides sizes. (**E**) Mouse epididymides stained with H&E (scale bar = 50 μm). (**F**) Mouse testicular cross sections stained with H&E (scale bar = 50 μm). Arrows: Leydig cells of the testes. (**G**) Weight of mouse epididymides during different recovery periods after cisplatin treatment. (**H**) Mouse testicular weight during different recovery periods after cisplatin treatment. (**I**) Detection of the testosterone level in the mouse testes. (**J**) Epididymal sperm count. (**K**) Detection of serum MDA in mice. (**L**) Detection of serum SOD activity in mice. (**M**) Detection of serum total antioxidant capacity in mice. (**N**) Detection of the serum melatonin level in mice. CP, cisplatin. Data are presented as the mean ± SEM. *N* = 6 mice per group. * *p* < 0.05, ** *p* < 0.01 versus the control groups.

To explore the effects of chemotherapeutic drugs on the testes, we constructed a mouse model using cisplatin. Mice were exposed to cisplatin and allowed to recover for different lengths of time, i.e., 8, 17, and 34 days. The weights of the mice are shown in Figure 2B. It was found that the testes and the epididymides were smaller than those of the control group (Figure 2C,D). HE staining showed that cisplatin not only disrupted the Leydig cells and spermatogonia, but also enlarged the space between the seminiferous tubules. Furthermore, cisplatin reduced the number of sperm in the epididymal duct. Moreover, no sperm was found in the testes and epididymides after cisplatin treatment and recovery for a spermatogenic cycle (34 days) (Figure 2E,F). After the mice were treated with cisplatin and allowed to recover for 8, 17, and 34 days, the weights of the testes and the epididymides decreased significantly (Figure 2G,H). Consistently, compared with the control group, cisplatin also caused a decrease in the testosterone content (Figure 2I). The sperm concentrations of the mice gradually decreased after cisplatin treatment and recovery for 8, 17, and 34 days (Figure 2J). To explore the oxidative stress and the melatonin effect on cisplatin-treated mice, the levels of MDA, SOD, melatonin, and T-AOC in the peripheral sera of mice were detected. Compared to the control group, the MDA level was significantly increased after cisplatin treatment and recovery for 8, 17, and 34 days (Figure 2K). The SOD, T-AOC, and melatonin levels were decreased after cisplatin treatment and recovery for 8, 17, and 34 days (Figure 2L–N). Collectively, our results confirm that the mouse testes and Leydig cells were damaged by cisplatin. Cisplatin caused oxidative stress in mice and reduced the levels of melatonin and testosterone in the testes. These injuries were highest after treatment with cisplatin and recovery for 17 days. Therefore, the testes of mice treated with cisplatin and recovered for 17 days were analyzed by iTRAQ quantitative proteomics.

### 3.3. iTRAQ Quantitative Proteomics of Mouse Testes and Expression of Oxidative-Stress-Related Proteins

**Figure 3:**
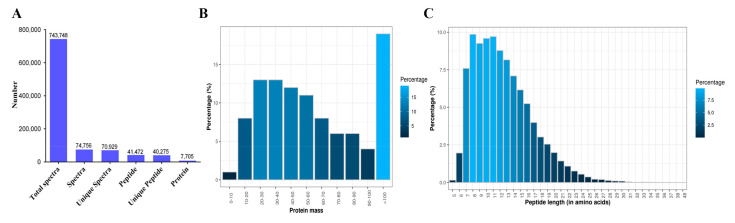

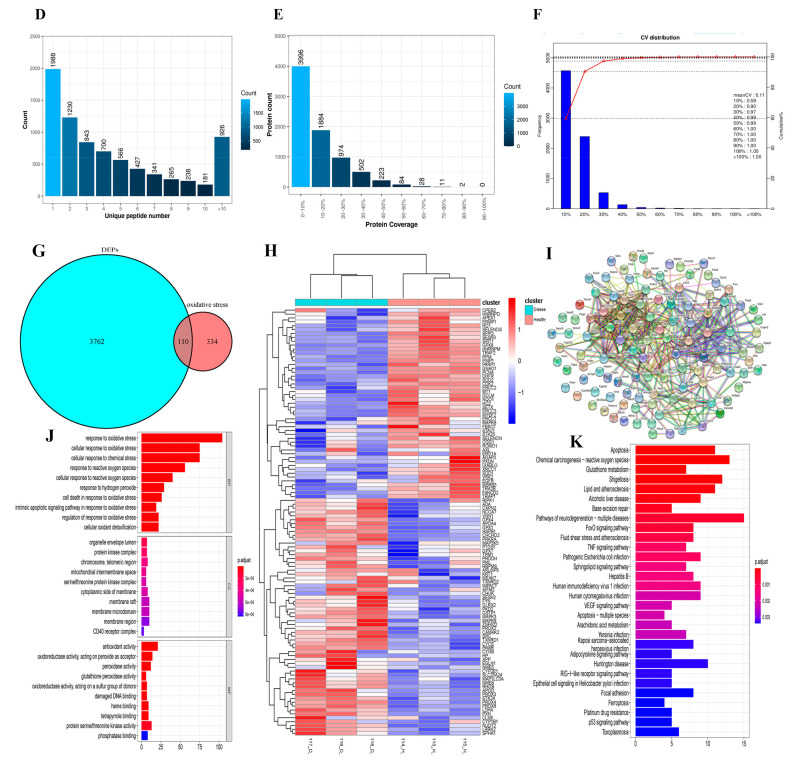
iTRAQ quantitative proteomics of mouse testes during recovery for 17 days after cisplatin/no treatment and the expression of oxidative-stress-related proteins in mice during different recovery periods after cisplatin/non-cisplatin treatment. (**A**) According to iTRAQ proteomic analysis, total spectra, spectra, unique spectra, peptides, unique peptides, and proteins were identified. (**B**) Proportional distribution of identified proteins based on their mass. (**C**) Peptide length distribution as a percentage of the total protein length. (**D**) Number of unique peptides that matched the proteins. (**E**) Protein coverage distribution in all of the identified proteins. (**F**) Three repetitions of the coefficient of variation distribution. (**G**) In the testes of the control d17 and CP d17 groups, a Venn diagram was used to identify the differentially expressed proteins associated with oxidative stress. (**H**) Heatmap showing the clustering of 110 DEPs between the control d17 and the CP d17 groups. (**I**) Connections among the 110 differentially expressed proteins relevant to oxidative stress revealed using the PPI network obtained from the STRING database. (**J**) The ordinate indicates the gene’s name, the abscissa shows the number of genes, and the color reflects the adjusted *p*-value in an enriched gene ontology (GO) analysis of the 110 DEPs linked to oxidative stress. (**K**) Enrichment KEGG pathway analysis of the 110 DEPs related to oxidative stress; the ordinate displays the pathway name, the abscissa displays the differential protein count, and the color represents the adjusted *p*-value.

In this study, iTRAQ was employed to assess the proteome changes between the control d17 group (healthy3, healthy5, and healthy7) and the CP d17 group (dis2, dis5, and dis8). As shown in Figure 3A, 743,748 spectra were generated and 41,472 peptides and 7705 proteins were identified. The protein mass distribution above 10 kDa covered 99%, among which 10−60 kDa covered 57%, 60−100 kDa comprised 24%, and above 100 kDa accounted for 19% (Figure 3B). About 65% of the peptides were 5–13 amino acids in length (Figure 3C). The unique peptide number distribution is shown in Figure 3D. The proteins with 1 peptide, 2–5 peptides, 6–10 peptides, and more than 11 peptides comprised 1988, 3339, 1452, and 926, respectively. A total of 7356 proteins were identified with sequence coverage ranging from 0% to 40%. However, only 4.47% of the proteins were identified with sequence coverage of >40% (Figure 3E). The coefficient of variation (CV), defined as the ratio of the standard deviation (SD) to the mean (CV = SD/mean), was used to quantitatively assess the repeatability. The lower the CV, the better the reproducibility. The CV distribution (mean CV: 0.11) for three replicates showed good reproducibility (Figure 3F). Differentially expressed proteins were screened according to the standard of fold-change values of > 1.1 or <0.9 and Q-values of < 0.05. Based on these criteria, 3862 differently expressed proteins (DEPs) between the control d17 group and CP d17 group were identified. In addition, 444 oxidative-stress-related genes were retrieved from GSEA: M3223. Upon comparing 3862 proteins with 444 oxidative-stress-related genes, 110 differentially expressed proteins showed a response to oxidative stress (Figure 3G). These DEPs were used to draw the cluster analysis diagram (Figure 3H), which intuitively reflected the expression differences between the control d17 group and the CP d17 group. The interaction network among 110 differentially expressed proteins predicted by the STRING database showed that SIRT1, SOD1, SOD2, NQO1, and HO-1 proteins may interact with MT1 (Figure 3I). A GO enrichment analysis of these 110 DEPs showed that the functions of these differentially expressed proteins could be analyzed from three aspects: biological process (BP), cellular component (CC), and molecular function (MF) (Figure 3J). BP mainly includes a response to oxidative stress, a cellular response to oxidative stress, a cellular response to chemical stress, and a response to reactive oxygen species. The molecular function mainly focuses on antioxidant activity, oxidoreductase activity, and peroxidase activity. The KEGG pathway enrichment analysis further indicated that these DEPs were mostly in chemical carcinogenesis reactive oxygen species, apoptosis, shigellosis, and pathways of neurodegeneration multiple diseases (Figure 3K). These GO and KEGG enrichment analysis results indicated that the DEPs were mostly focused on oxidative stress or reactive oxygen species between the control d17 group and the CP d17 group.

### 3.4. Cisplatin Attenuates the Expression of SIRT1/Nrf2 Signaling at Different Recovery Times

**Figure 4:**
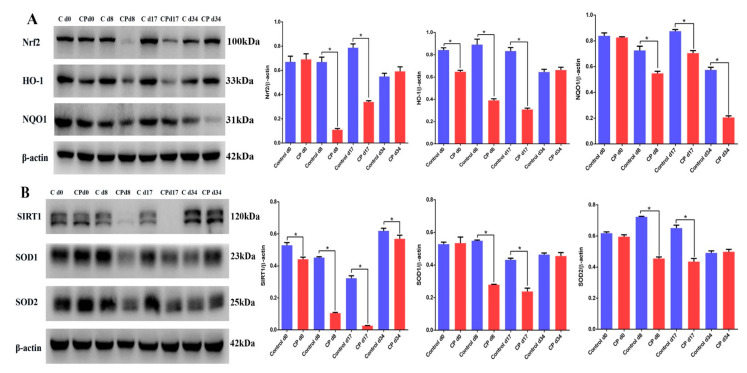
Cisplatin attenuates the expression of SIRT1/Nrf2 signaling at different recovery times. (**A**) Determination of Nrf2, HO-1, and NQO1 protein levels performed by Western blotting in mouse testes from the different groups. The blots were analyzed using Image J software. (**B**) Determination of the SIRT1, SOD1, and SOD2 protein levels performed by Western blotting in mouse testes from the different groups. The blots were analyzed using Image J software. CP, cisplatin. Data are presented as the mean ± SEM (*N* = 3–6 per group). * *p* < 0.05 compared with controls.

To further confirm that cisplatin would lead to the downregulation of the MT1/MT2 and SIRT1/Nrf2 signaling pathways, mice were treated with cisplatin and allowed to recover for 8, 17, and 34 days. The immunofluorescence results showed that MT1 and MT2 were located in Leydig cells and highly expressed in the Leydig cells of mouse testes (Figure S2A,B). The negative control results of MT1 and MT2 immunofluorescence in mouse testicular tissue are shown in Figure S1B. The Western blotting results showed that the expression levels of Nrf2, HO-1, NQO1, SIRT1, SOD1, and SOD2 in mouse testes were significantly lower than those of the control groups after the mice were treated with cisplatin and recovered for 8, 17, and 34 days (Figure 4A,B). Similarly, the results of cluster analysis showed that the expression levels of the Nrf2, HO-1, NQO1, SIRT1, SOD1, and SOD2 proteins were lower than those in the control d17 group. These results indicate that cisplatin can cause the downregulation of Nrf2 and SIRT1 signaling in mouse testes.

### 3.5. Melatonin Can Alleviate the Damage to Mouse Testes and LEYDIG Cells Caused by Cisplatin

**Figure 5:**
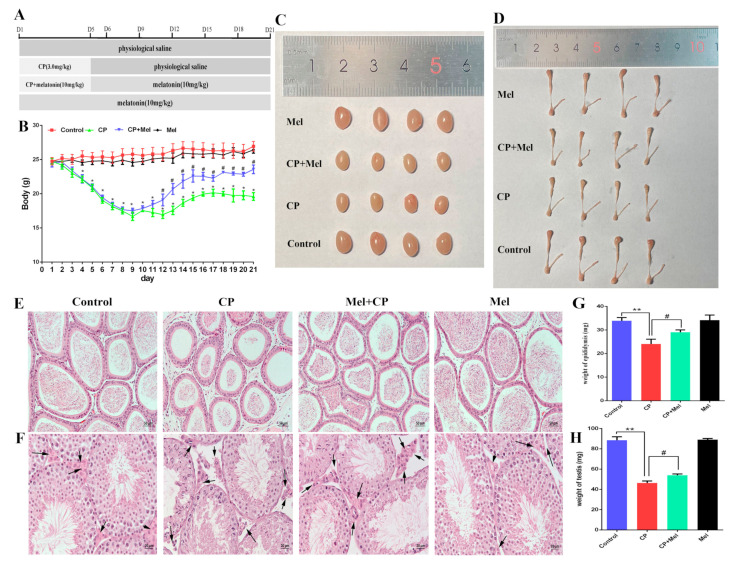

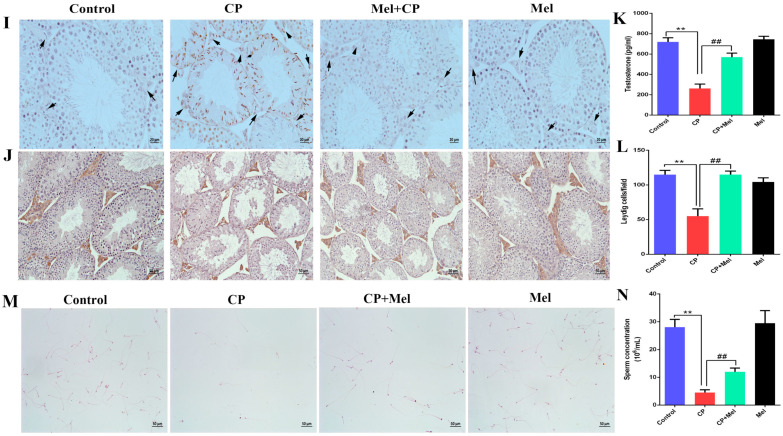
Melatonin can alleviate damage to mouse testes and Leydig cells induced by cisplatin. (**A**) Experimental design. (**B**) Changes in the body weight of mice. (**C**) Images of mouse testes sizes. (**D**) Images of mouse epididymides sizes. (**E**) Mouse epididymides stained with H&E (scale bar = 50 μm). (**F**) Mouse testicular cross sections stained with H&E (scale bar = 50 μm). (**G**) Testicular cross sections stained with TUNEL to show apoptosis (scale bar = 50 μm). (**H**) 3β-HSD staining was used to immunolocalize Leydig cells. (**I**) Number of Leydig cells. (**J**) H&E-stained mouse sperm (scale bar = 50 μm). (**K**) Mouse testicular weights. (**L**) Mouse epididymides weights. (**M**) Testosterone level in mouse testes. (**N**) Epididymal sperm cells were counted. CP, cisplatin. CP + Mel, cisplatin + melatonin. Mel, melatonin. Data are presented as the mean ± SEM. (*N* = 8 per group). * *p* < 0.05, ** *p* < 0.01 compared with controls. # *p* < 0.05, ## *p* < 0.01 compared with the CP group.

To reduce the damage to male testes caused by cisplatin, mice were treated with melatonin and cisplatin, as shown in Figure 5A. Compared with the cisplatin-treated group, the weight of the mice in the CP + Mel group increased significantly (Figure 5B), and the testes and epididymides from the melatonin-pretreated animals were larger than those form the CP group (Figure 5C,D). Compared with the CP group, the epididymal tubes of the other groups were full of sperm in the control group, the CP + Mel group, and the Mel group (Figure 5E). The control group, the CP + Mel group, and the Mel group showed that the histological structures of the seminiferous tubules were normal, the germ cells were arranged in different differentiation stages, the morphology of the Leydig cells was normal, and there were many sperm cells in the lumen (Figure 5F). Consistently, the weights of the epididymides and testes in the CP + Mel group were also significantly higher than those in the CP group (Figure 5G,H). Melatonin significantly alleviated the damage to testicular DNA in the CP + Mel group (Figure 5I). Melatonin could also increase the number of sperm cells (Figure 5M). Moreover, melatonin could significantly alleviate the reduction of the testosterone content and sperm concentration in the CP + Mel group (Figure 5K,N). We used 3β-HSD to immunostain and count the Leydig cells. It was found that cisplatin reduced the number of Leydig cells and melatonin could protect Leydig cells from cisplatin (Figure 5J,L). Overall, melatonin inhibits the cisplatin-induced damage to testes and sperm cells in mice.

### 3.6. Melatonin Can Attenuate Oxidative Stress Caused by Cisplatin in Mouse Sera and Testes

**Figure 6:**
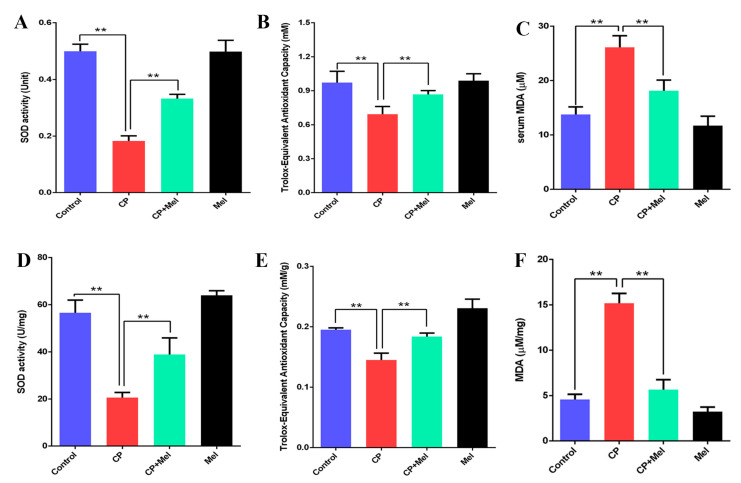
Melatonin can attenuate oxidative stress caused by cisplatin in mouse sera and testes. (**A**) Detection of serum SOD activity in mice. (**B**) Detection of serum total antioxidant capacity in mice. (**C**) Detection of serum MDA in mice. (**D**) Detection of SOD activity in mouse testes. (**E**) Detection of the total antioxidant capacity in mouse testes. (**F**) Detection of MDA in mouse testes. CP, cisplatin. CP + Mel, cisplatin + melatonin. Mel, melatonin. Data are presented as the mean ± SEM. (*N* = 8 per group). ** *p* < 0.01 compared with controls or CP groups.

To determine whether melatonin inhibited oxidative stress, the oxidative-stress markers SOD and T-AOC in the sera and testes of the melatonin-pretreated mice were examined. The SOD activity and T-AOC increased significantly in the CP + Mel group and Mel group (Figure 6A,B,D,E). Conversely, the MDA levels in the sera and testes were significantly decreased in the CP + Mel group and Mel group (Figure 6C,F). As mentioned above, melatonin inhibits the cisplatin-induced oxidative stress and the damage to testes and sperm cells in mice.

### 3.7. Melatonin Could Attenuate the Downregulation of MT1, MT2, and SIRT1/Nrf2 Antioxidant Signaling by Cisplatin in Mouse Testes

**Figure 7:**
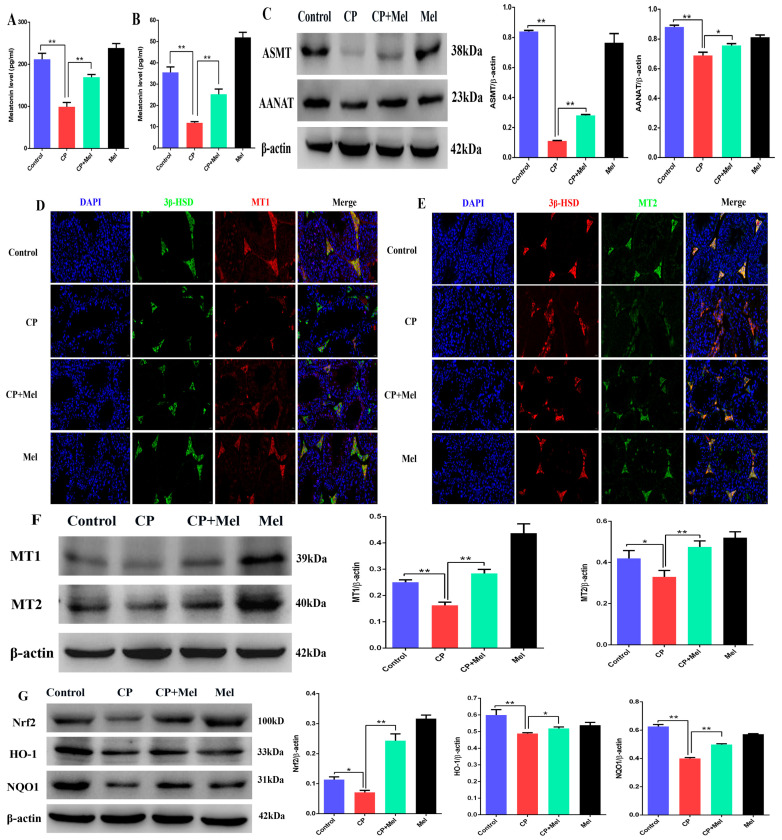

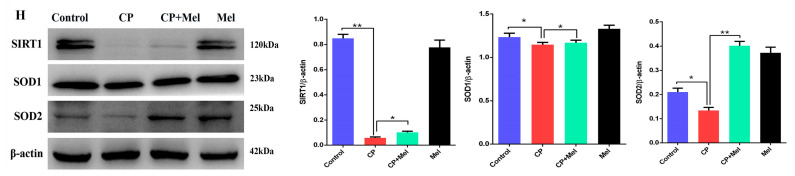
Melatonin could attenuate the downregulation of MT1, MT2, and SIRT1/Nrf2 antioxidant signaling by cisplatin in mouse testes. (**A**) Melatonin level in mouse serum. (**B**) Melatonin level in mouse testes. (**C**) Determination of the ASMT and AANAT protein levels was performed by Western blotting in mouse testes. The blots were quantified using Image J software. (**D**) MT1 immunofluorescence in mouse testicular tissue. 3β-HSD was tagged with green fluorescence, while MT1 was tagged with red fluorescence. The nucleus was labeled with DAPI (scale bar = 20 μm). (**E**) MT2 immunofluorescence in mouse testicular tissue. 3β-HSD was tagged with red fluorescence, while MT2 was tagged with green fluorescence. The nucleus was labeled with DAPI (scale bar = 20 μm). (**F**) Determination of the MT1 and MT2 protein levels was performed by Western blotting in mouse testes. The blots were quantified using Image J software. (**G**) Determination of Nrf2, HO-1, and NQO1 protein levels was performed by Western blotting in mouse testes. The blots were quantified using Image J software. (**H**) Determination of SIRT1, SOD1, and SOD2 protein levels was performed by Western blotting in mouse testes. The blots were quantified using Image J software. CP, cisplatin. CP + Mel, cisplatin + melatonin. Mel, melatonin. Data are presented as the mean ± SEM (*N* = 8 per group). * *p* < 0.05 compared with controls or CP groups. ** *p* < 0.01 compared with controls or CP groups.

Upon further studying the levels of melatonin and the melatonin-mediated signaling pathway, we found that the melatonin level decreased (compared to that in the control group) in the testes and sera of mice treated with cisplatin. However, compared with the CP group alone, pretreatment with melatonin markedly increased the level of melatonin (Figure 7A,B). Furthermore, melatonin pretreatment significantly alleviated the downregulation of ASMT and AANAT expression (compared to the CP group) in the testes (Figure 7C). Interestingly, the immunofluorescence results for the testicular tissue showed that MT1 and MT2 proteins were mainly located in the testicular Leydig cells in the CP group. MT1 and MT2 fluorescence increased significantly in the testes of the melatonin pretreatment mice (Figure 7D,E). Compared with the CP group, melatonin significantly alleviated the downregulation of MT1 and MT2 in testicular tissue under treatment with cisplatin (Figure 7F). To investigate whether melatonin alleviates the cisplatin-induced downregulation of the Nrf2 and SIRT1 signaling pathways, the protein levels of Nrf2, HO-1, NQO1, SIRT1, SOD1, and SOD2 were measured. The levels of these proteins in the cisplatin-treated mouse testes were significantly lower than those in the control group. The cisplatin-induced downregulation of the Nrf2 and SIRT1 signaling pathways was reversed by melatonin treatment (Figure 7G,H). These results indicate that melatonin can alleviate the downregulation of melatonin-synthesizing enzymes, MT1, MT2, and the SIRT1/Nrf2 signaling pathways by cisplatin.

### 3.8. Cisplatin Increased Apoptosis and Downregulated MT1, MT2, and SIRT1/Nrf2 Signaling Pathways in Leydig Cells

**Figure 8:**
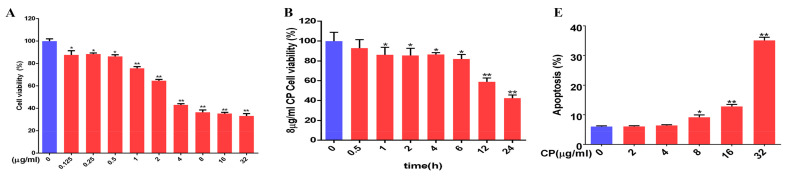

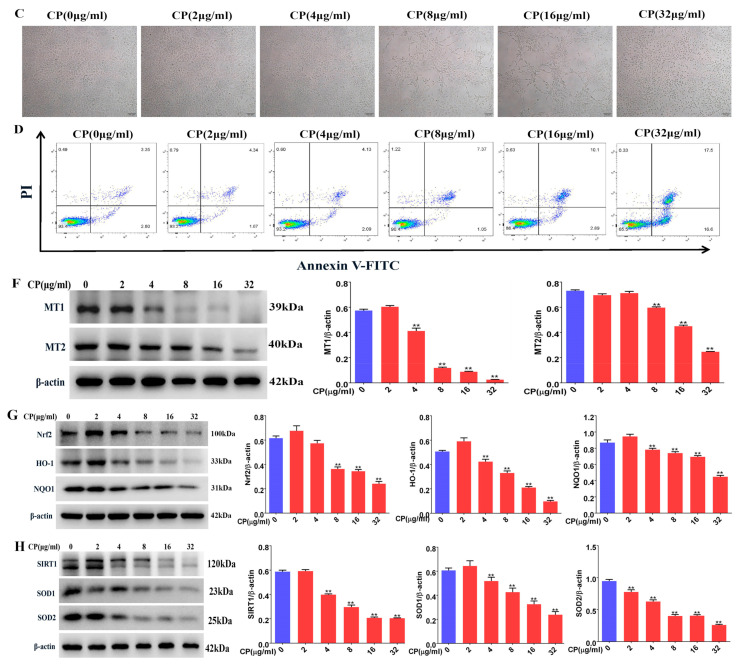
Cisplatin increased apoptosis and downregulated the MT1, MT2, and SIRT1/Nrf2 signaling pathways in Leydig cells. (**A**) CCK8 was used to determine the relative cell survival of Leydig cells after treatment with various doses of cisplatin for 24 h. (**B**) CCK8 was used to determine the relative cell viability of Leydig cells treated with 8 μg/mL of cisplatin for different lengths of time. (**C**) Images of Leydig cells treated with various doses of cisplatin for 24 h. (**D**) Apoptosis was observed by flow cytometry analysis in Leydig cells treated with various doses of cisplatin for 24 h. (**E**) Apoptotic cell percentages are shown. (**F**) Determination of MT1 and MT2 protein levels was performed by Western blotting in Leydig cells treated with various doses of cisplatin for 24 h. The blots were quantified using Image J software. (**G**) Determination of the Nrf2, HO-1, and NQO1 protein levels was performed by Western blotting in Leydig cells treated with various doses of cisplatin for 24 h. The blots were quantified using Image J software. (**H**) Determination of SIRT1, SOD1, and SOD2 protein levels was performed by Western blotting in Leydig cells treated with various doses of cisplatin for 24 h. The blots were quantified using Image J software. CP, cisplatin. Data are presented as the mean ± SEM (*N* = 4 per group). * *p* < 0.05 or ** *p* < 0.01 compared with controls.

Animal experiments showed that cisplatin could cause apoptosis and decrease the number of Leydig cells in the testes. To explore the effects of different concentrations of cisplatin on testicular Leydig cells in vitro, we isolated and cultured primary Leydig cells from mouse testes. The cells were treated with different concentrations of cisplatin (0–32 μg/mL) for 24 h. The results showed that the cell viability decreased in a concentration-dependent manner (Figure 8A). An optical microscopic examination showed that some cells shrank and the cell-to-cell space became larger in the Leydig cells with cisplatin (8 and 16 μg/mL) treatment. The Leydig cells treated with 32 μg/mL cisplatin became round and floated (Figure 8C). Therefore, we chose 8 μg/mL cisplatin to treat Leydig cells for different lengths of time, and the cell viability decreased gradually with the prolongation of treatment time (Figure 8B). To ascertain the effect of cisplatin-induced Leydig cell apoptosis, the apoptosis levels were detected by flow cytometry. As shown in Figure 8D,E, cisplatin treatment for 24 h increased the number of apoptotic cells. Meanwhile, cisplatin attenuated the expression of MT1, MT2, Nrf2, and SIRT1 signaling in Leydig cells (Figure 8F–H).

### 3.9. Leydig Cells Failed to Self-Recovery after Removing Cisplatin

**Figure 9:**
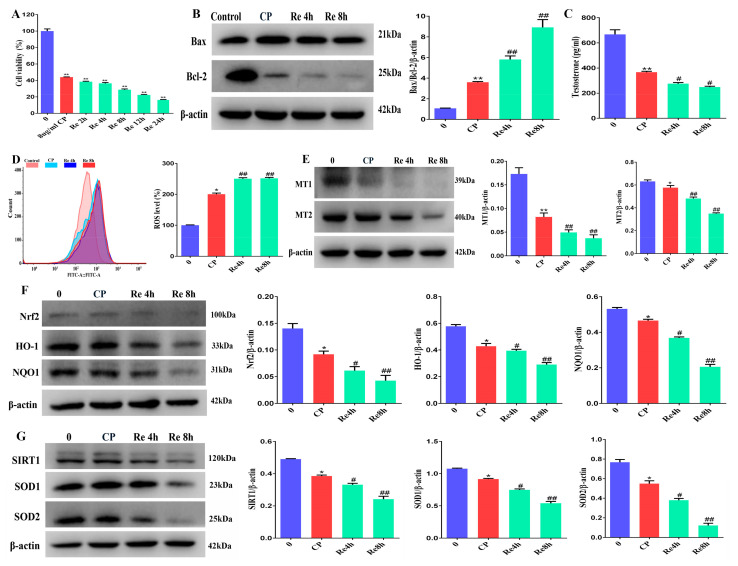
Leydig cells failed to self recover after removing cisplatin. (**A**) CCK8 was used to determine the relative cell viability of Leydig cells treated with 8 μg/mL cisplatin and removal of cisplatin recovery for 2 h, 4 h, 8 h, 12 h, and 24 h. (**B**) Determination of Bcl-2 and Bax protein levels was performed by Western blotting in Leydig cells treated with 8 μg/mL of cisplatin and removal of cisplatin recovery for 4 h and 8 h. The blots were quantified using Image J software. (**C**) Content of testosterone in Leydig cells treated with 8 μg/mL of cisplatin and removal of cisplatin recovery for 4 h and 8 h. (**D**) Flow cytometry was used to measure the concentrations of reactive oxygen species in Leydig cells treated with 8 μg/mL of cisplatin and removal of cisplatin recovery for 4 h and 8 h; ROS were detected by DCFH-DA staining. (**E**) Determination of MT1 and MT2 protein levels was performed by Western blotting in Leydig cells treated with 8 μg/mL cisplatin and removal of cisplatin recovery for 4 h and 8 h. The blots were quantified using Image J software. (**F**) Determination of Nrf2, HO-1, and NQO1 protein levels was performed by Western blotting in Leydig cells treated with 8 μg/mL cisplatin and removal of cisplatin with recovery for 4 h and 8 h. The blots were quantified using Image J software. (**G**) Determination of SIRT1, SOD1, and SOD2 protein levels was performed by Western blotting in Leydig cells treated with 8 μg/mL cisplatin and removal of cisplatin with recovery for 4 h and 8 h. The blots were quantified using Image J software. CP, cisplatin. Re, recovery. Data are presented as the mean ± SEM (*N* = 4 per group). * *p* < 0.05 or ** *p* < 0.01 compared with controls. # *p* < 0.05 or ## *p* < 0.01 compared with CP groups.

To explore whether Leydig cells underwent self recovery after the removal of cisplatin in vitro. The cells were treated with 8 μg/mL of cisplatin and allowed to recover for 2 h, 4 h, 8 h, 12 h, and 24 h after the removal of cisplatin. We found that the cell viability decreased further (Figure 9A). We also determined the expressions of Bcl-2, an anti-apoptotic protein, and Bax, a pro-apoptotic factor. The results revealed that the levels of Bax in Leydig cells treated with cisplatin were substantially greater than those in the control cells, whereas the Bcl-2 levels were much lower (Figure 9B). Testosterone is secreted by Leydig cells, and the testosterone levels decreased in the cisplatin-treated group and the recovery group (Figure 9C). Correspondingly, the levels of ROS in Leydig cells in the recovery group were significantly lower than those in the cisplatin-treated and control groups (Figure 9D). As presented in Figure 9E, the protein levels of MT1 and MT2 in Leydig cells were significantly decreased in the cisplatin-treated and recovery groups. Compared with the cisplatin group, the protein levels of Nrf2, HO-1, NQO1, SIRT1, SOD1, and SOD2 decreased further in the recovery groups (Figure 9F,G). Based on the above results, we found that 8 μg/mL cisplatin induced slight oxidative damage in Leydig cells. We chose Leydig cells treated with 8 μg/mL cisplatin for a follow-up experiment to study the protective mechanism of melatonin.

### 3.10. Melatonin Could Attenuate the Downregulation of the MT1, MT2, and SIRT1/Nrf2 Signaling Pathways by Cisplatin in Leydig Cells

**Figure 10:**
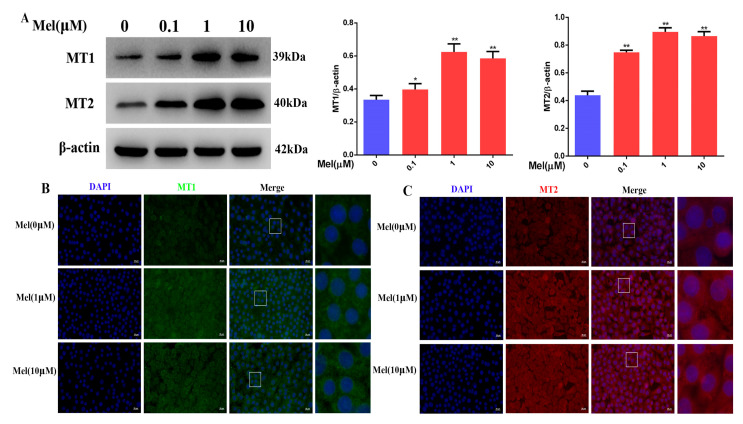

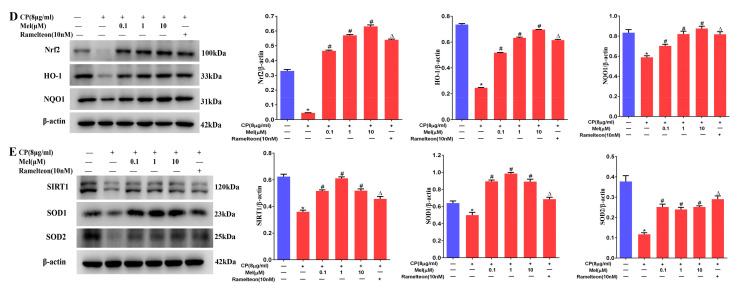
Melatonin could attenuate the downregulation of the MT1, MT2, and SIRT1/Nrf2 signaling pathways by cisplatin in Leydig cells. (**A**) Determination of MT1 and MT2 protein levels was performed by Western blotting in Leydig cells treated with different concentrations of melatonin for 24 h. The blots were quantified using Image J software. (**B**) MT1 immunofluorescence in Leydig cells treated with 1 μM of melatonin. MT1 was tagged with green fluorescence. The nucleus was labeled with DAPI (scale bar = 20 μm). (**C**) MT2 immunofluorescence in Leydig cells treated with 1 μM of melatonin. MT1 was tagged with red fluorescence. The nucleus was labeled with DAPI (scale bar = 20 μm). (**D**) Determination of Nrf2, HO-1, and NQO1 protein levels was performed by Western blotting in cisplatin-induced Leydig cells pretreated with different concentrations of melatonin or ramelteon. The blots were quantified using Image J software. (**E**) Determination of the SIRT1, SOD1, and SOD2 protein levels was performed by Western blotting in cisplatin-induced Leydig cells pretreated with different concentrations of melatonin or ramelteon. The blots were quantified using Image J software. CP, cisplatin. Mel, melatonin. Data are presented as the mean ± SEM (*N* = 4 per group). * *p* < 0.05 or ** *p* < 0.01 compared with controls. # *p* < 0.05 compared with CP groups. ∆ *p* < 0.05 compared with CP groups.

To determine whether melatonin alleviates the cisplatin-induced downregulation of the MT1, MT2, and SIRT1/Nrf2 signaling pathways in Leydig cells, our experimental data showed that melatonin (0.1, 1, and 10 μM) increased the expression of the MT1 and MT2 receptor proteins and fluorescence signal in Leydig cells (Figure 10A–C). Meanwhile, melatonin (0.1, 1, and 10 μM) attenuated cisplatin’s downregulation of the protein expression of Nrf2, HO-1, and NQO1 in Leydig cells (Figure 10D). Expectedly, melatonin (0.1, 1, and 10 μM) also markedly alleviated the cisplatin-induced reduction of the SIRT1, SOD1, and SOD2 protein levels in Leydig cells (Figure 10E). Thus, melatonin could attenuate the downregulation of the MT1, MT2, and SIRT1/Nrf2 signaling pathways by cisplatin in Leydig cells.

### 3.11. Melatonin Protects Leydig Cells from Cisplatin-Induced Oxidative Damage by Activating the MT1/MT2-Mediated SIRT1/Nrf2 Signaling Pathway

**Figure 11:**
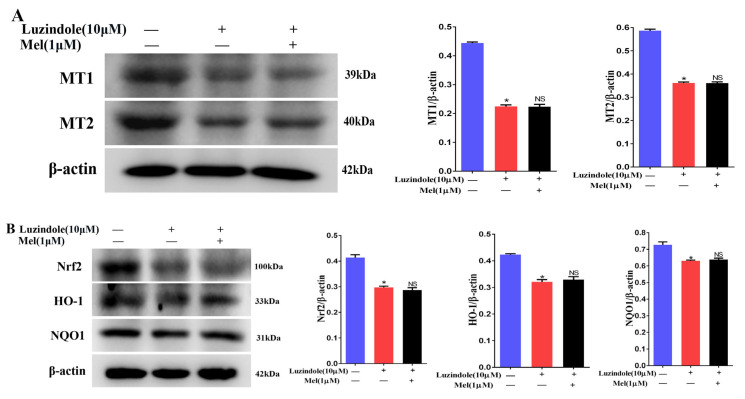

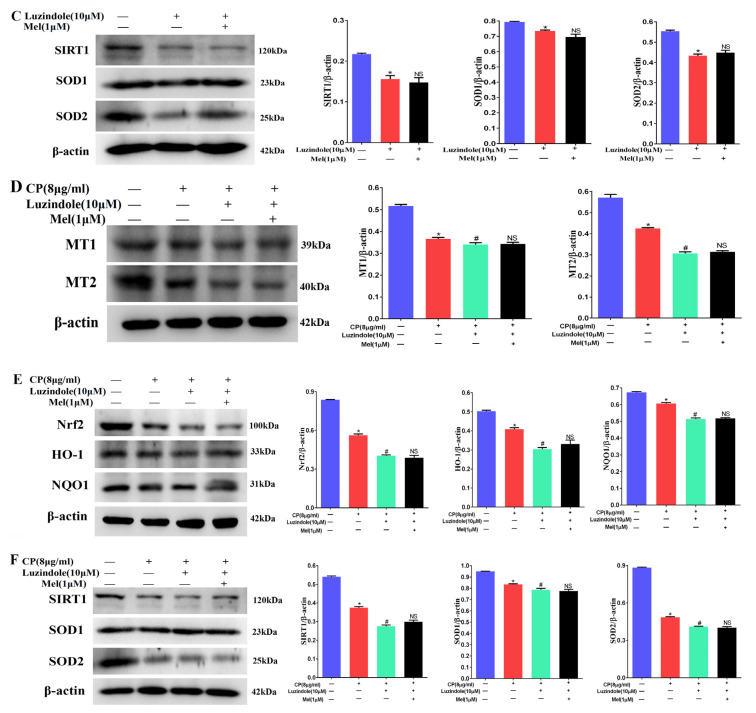

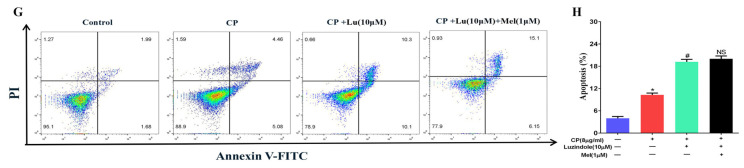
Melatonin protects Leydig cells from cisplatin-induced oxidative damage by activating the MT1/MT2-mediated SIRT1/Nrf2 signaling pathway. (**A**) Determination of MT1 and MT2 protein levels was performed by Western blotting in Leydig cells treated with Luzindole or Luzindole and melatonin. The blots were quantified using Image J software. (**B**) Determination of Nrf2, HO-1, and NQO1 protein levels was performed by Western blotting in Leydig cells treated with Luzindole or Luzindole + melatonin. The blots were quantified using Image J software. (**C**) Determination of SIRT1, SOD1, and SOD2 protein levels was performed by Western blotting in Leydig cells treated with Luzindole or Luzindole + melatonin. The blots were quantified using Image J software. (**D**) Determination of MT1 and MT2 protein levels was performed by Western blotting in control, CP, Luzindole + CP, and Luzindole + melatonin + CP groups. The blots were quantified using Image J software. (**E**) Determination of Nrf2, HO-1, and NQO1 protein levels was performed by Western blotting in control, CP, Luzindole + CP, and Luzindole + melatonin + CP groups. The blots were quantified using Image J software. (**F**) Determination of SIRT1, SOD1, and SOD2 protein levels was performed by Western blotting in control, CP, Luzindole + CP, and Luzindole + melatonin + CP groups. The blots were quantified using Image J software. (**G**) Flow cytometry was used to detect apoptosis in control, CP, Luzindole + CP, and Luzindole + melatonin + CP groups. (**H**) Percentages of the apoptotic cells are shown. CP, cisplatin. Mel, melatonin. NS, no significance. Data are presented as the mean ± SEM (*N* = 4 per group). * *p* < 0.05 compared with controls. # *p* < 0.05 compared with CP groups. NS compared with Luzindole groups or CP + Luzindole groups.

We have confirmed that cisplatin can reduce the expression of MT1 and MT2 in mouse Leydig cells. To obtain more evidence that melatonin induced the SIRT1/Nrf2 signaling pathway via the MT1 and MT2 receptors, Leydig cells were treated with the MT1- and MT2-receptor blocker Luzindole or Luzindole and melatonin. As shown in Figure 11A, Luzindole significantly inhibited the expression of the MT1 and MT2 receptors. Luzindole treatment markedly downregulated the expression of Nrf2, NQO1, and HO-1 (Figure 11B). Similarly, Luzindole decreased the protein expression of SIRT1, SOD1, and SOD2 (Figure 11C). However, melatonin did not change the downregulation of the SIRT1/Nrf2 signaling pathway caused by Luzindole. Interestingly, Luzindole pretreatment apparently enhanced the effect of cisplatin in reducing the MT1, MT2, and SIRT1/Nrf2 signaling protein levels in Leydig cells (Figure 11D–F). Furthermore, the flow cytometry results showed that Luzindole aggravated Leydig cell apoptosis in the Lu + Cd group (Figure 11G,H). Overall, our results further verify that MT1 and MT2 mediate SIRT1/Nrf2 signaling in Leydig cells.

## 4. Discussion

The anticancer activity of cisplatin was accidentally discovered more than 50 years ago [51]. People have a deep understanding of the chemical and biochemical changes leading to its medicinal properties. It has helped to improve treatment schemes and develop second- and third-generation platinum chemotherapeutic drugs, such as carboplatin and oxaliplatin [12]. Carboplatin and oxaliplatin have lower ototoxicity than cisplatin [52]. Platinum chemotherapeutic drugs mainly work by binding to the DNA of tumor cells and interfering with its replication [53].

Furthermore, platinum chemotherapeutic drugs can cause excessive ROS production [54]. In the current study, it was found that chemotherapy-induced testicular tissue changes, including Leydig cell disruption, may be caused by oxidant/antioxidant imbalances [55]. Moreover, chemotherapy has a long-lasting effect on Leydig cells in cancer patients [56]. Hypothalamic neurons secrete GnRH, which triggers the release of LH and FSH from the pituitary into the peripheral circulation. The main function of LH is to stimulate Leydig cells to secrete testosterone [57]. Therefore, Leydig cell damage may lead to the abnormal secretion of testosterone. More and more studies have reported that platinum chemotherapeutic drugs can cause oxidative stress and abnormal gonadal hormone secretion [58,59]. Tayyaba et al. found that cisplatin decreased the levels of testosterone, LH, and FSH, decreased antioxidant enzyme activity, and increased the levels of oxidative stress [60]. A study reported that the testosterone levels decreased in 60–64% of rectal cancer patients, LH increased significantly in 80–91% of patients, FSH increased significantly in 95% of patients after chemotherapy, and the FSH values further increased in all patients during the one-year follow-up [11]. Similarly, in our study, it was also found that the serum testosterone levels decreased and the LH and FSH levels increased in gastrointestinal tumors patients with cisplatin or oxaliplatin treatment. However, there was no difference in the GnRH levels.

CP causes testicular toxicity, leading to oxidative stress, sperm toxicity, and DNA damage, resulting in infertility [61]. Numerous studies indicated that cisplatin significantly decreased the number of spermatogonia, Leydig cells, and Sertoli cells, the testicular volume, the height of the germinal epithelium, the number of Bcl-2-immunopositive cells, CAT, GSH, and SOD enzyme activities, and serum testosterone levels in the testes of rats. In contrast, the numbers of caspase-3-, Bax-, and 8-OHdG-immunepositive cells and MDA levels were increased in the testes of cisplatin-treated rats [16,55]. Interestingly, our results also demonstrated that the SOD activity and antioxidant capacity decreased and the content of MDA increased in the cisplatin-treated mice. The SOD activity and antioxidant capacity were still low, and the content of MDA further increased after the mice were treated with cisplatin and recovered for 8, 17, and 34 days. In addition, busulfan significantly reduced the testosterone levels [21,62]. Busulfan causes oxidative stress by reducing the expression of SOD2 and increasing intracellular reactive oxygen species [63]. Busulfan induced spermatogonia apoptosis and increased extracellular signal-regulated kinase (ERK) and p38 phosphorylase-activation by upregulating caspase-3 and downregulating SIRT1 [25]. Similarly, our results confirmed that cisplatin injured testicular tissue and downregulated the expression of the anti-oxidant-stress proteins SIRT1, SOD1, and SOD2. Furthermore, the expression of the Nrf2, HO-1, and NQO1 proteins decreased after mice were treated with cisplatin and recovered for 8, 17, and 34 days. In addition, we also found that cisplatin reduced the levels of melatonin and testosterone in humans and mice sera. Cisplatin also reduced the melatonin levels in the testes. Exogenous supplementation with melatonin can effectively alleviate the damage by cisplatin in mouse testes. Therefore, protection against cisplatin-induced testicular tissue injury is still a key issue.

Melatonin and its derivatives are potent antioxidants and directly scavenge free radicals [64]. Melatonin possesses anti-aging, anti-inflammatory, and anti-apoptotic capabilities, as well as immunity-enhancing and cancer-fighting characteristics [65,66,67]. Melatonin also has a significant function in male reproduction by regulating the release of steroid hormones. Melatonin protects the testes against high temperatures, environmental chemicals, and medicines. Melatonin has beneficial effects on the reproductive process through receptor-dependent and receptor-independent pathways. Its receptor-independent action is attributed to its free-radical-scavenging behavior, whereas its receptor-dependent activity is attributed to its nuclear receptors (ROR-alpha) or membrane receptors (MT1 and MT2) [27]. A previous study showed that non-selective MT1-/MT2-receptor antagonists could inhibit the anti-apoptotic effect of melatonin in cisplatin-treated mice ovaries. In contrast, selective MT2-receptor antagonists did not change the anti-apoptotic effect, indicating that melatonin plays an anti-oxidant and anti-apoptotic role in mouse ovaries through the MT1 receptor [23]. In another study, MT1 and MT2 were shown to be highly expressed in the Leydig cells of male Kunming White outbred strain mice [32]. Melatonin induces a two-fold increase in MT1-receptor expression and a four-fold increase in MT2-receptor expression in mouse ovaries. Therefore, melatonin has a very important regulatory effect on reproduction [68]. Zhang et al. found that female mice with MT1 knockout (mt1-ko) showed a significant decrease in the number of oocytes and litter size. The expressions of SIRT1, c-myc, and chop were downregulated in the ovaries of mt1-ko mice [35]. In addition, it was found that MT1 and MT2 knockout promoted the expression of p53, caspase-3, and Bcl-2 in mltc-1 cells treated with hCG. Therefore, MT1 and MT2 were essential to inhibit hCG-induced endoplasmic reticulum stress and apoptosis [32]. Melatonin significantly reduced the tumor size and microvessel density in ovarian cancer rats. Moreover, the level of serum melatonin and the expression of MT1 increased significantly after melatonin treatment [69]. Melatonin promotes SIRT1 and nuclear transcription expression through melatonin receptors. Furthermore, melatonin prevents Palmitic acid (PA)-induced ROS production and mitochondrial dysfunction through the SIRT1 signaling pathway in type-B spermatogonial stem cells [70]. We wanted to elucidate whether melatonin could regulate the SIRT1 signaling pathway through the melatonin receptors and alleviate the damage to the testes and Leydig cells caused by cisplatin. In the present study, we found that MT1 and MT2 receptors were mainly expressed in mouse Leydig cells. The expression of melatonin receptors (MT1 and MT2) and SIRT1 was reduced in cisplatin-treated mice and Leydig cells. In addition, the expression of the SIRT1, SOD1, and SOD2 proteins was decreased in cisplatin-treated mice and Leydig cells. However, melatonin pretreatment alleviated cisplatin-induced oxidative stress and testicular damage. Melatonin also attenuated the cisplatin-induced downregulation of SIRT1 signaling.

Notably, the Nrf2 pathway plays an important role in preventing oxidative stress. Oxidative stress stimulates the translocation of Nrf2 from the cytoplasm to the nucleus, where it binds to antioxidant-response elements (AREs) and promotes the expression of many genes encoding antioxidant enzymes, such as HO-1 and NQO1 [71]. Nrf2 regulates the transcriptional activity of endogenous antioxidant enzymes, including SOD, CAT, and GPX. The Nrf2 antioxidant response element pathway is an important system for preventing oxidative stress [72]. Several studies indicated that H_2_O_2_ caused oxidative stress and lowered the protein levels of Nrf2, HO-1, and NQO1 in rooster Leydig cells [73]. Melatonin stimulated the production of Nrf2, HO-1, and NQO1 in H_2_O_2_-exposed rooster Leydig cells. Therefore, Nrf2 activation is critical for the protective effect of melatonin. Melatonin reduces oxidative stress and apoptosis by promoting the Nrf2/HO-1 signaling pathway in mouse testicular cells. Melatonin can also inhibit stress-induced spermatogenic injury [74]. Melatonin inhibits hyperhomocysteinemia-induced oxidative stress and apoptosis in rat smooth muscle cells via the Nrf2/HO-1 pathway [75]. Guo et al. found that psychological stress can induce oxidative stress and apoptosis in testicular cells. Melatonin ameliorates oxidative stress and apoptosis by upregulating the NF-κB/iNOS and Nrf2/HO-1 signaling pathways [76]. We also found that oxidative damage was elevated in the testes and sera of cisplatin-treated mice. Compared with the cisplatin treatment group, melatonin pretreatment increased the expression of the Nrf2, NQO1, and HO-1 proteins in mouse testis. Melatonin has a protective effect on cisplatin-induced oxidative stress. A recent study found that melatonin protected human sperm by activating Nrf2 and its downstream gene HO-1 [77]. Melatonin exerted its anti-apoptotic and anti-oxidative effects by activating the SIRT1 signal pathway in mouse Leydig cells [78]. However, it was unclear whether melatonin could inhibit cisplatin-induced oxidative damage to Leydig cells by activating the MT1/MT2-mediated SIRT1/Nrf2 signaling pathway. Our results show that melatonin can increase the expression of MT1 and MT2 in Leydig cells. Melatonin can alleviate the cisplatin-induced downregulation of SIRT1/Nrf2 signaling in Leydig cells. The treatment of Leydig cells with a melatonin-receptor antagonist (Luzindole) decreased the expression of MT1/MT2 and SIRT1/Nrf2 signaling. Melatonin did not alleviate the decrease of SIRT1/Nrf2 signal in Leydig cells treated with Luzindole. Moreover, inhibiting the melatonin receptor increased the cisplatin-induced apoptosis of Leydig cells. Pretreatment with Luzindole can aggravate cisplatin’s effects on the SIRT1/Nrf2 signaling pathway. This suggests that melatonin may protect the testes and Leydig cells from cisplatin-induced oxidative stress by activating the MT1/MT2-mediated SIRT1/Nrf2 signaling pathway.

## 5. Conclusions

**Figure 12:**
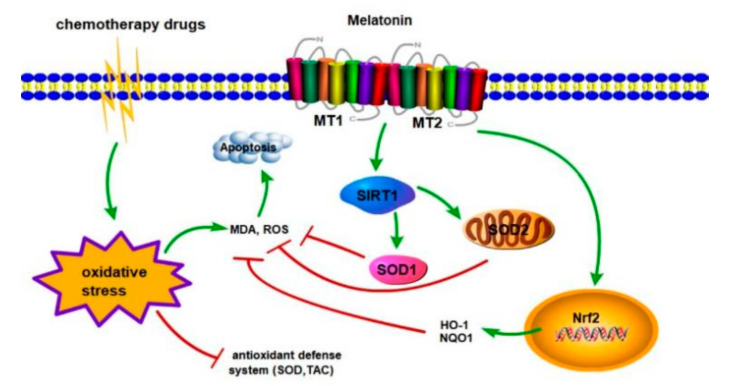
Schematic diagram illustrating the mechanism by which melatonin regulates the SIRT1/Nrf2 pathway through the melatonin receptors (MT1/MT2).

This study mainly investigated the changes in gonadal hormones, oxidative stress, and melatonin levels in gastrointestinal tumor patients and mice treated with platinum chemotherapeutic drugs. Additionally, the expression and localization of melatonin receptors were studied in gastrointestinal tumor patients with azoospermia caused by platinum chemotherapeutic drugs. Whether melatonin can protect mouse testes and Leydig cells from cisplatin-induced injury by activating the MT1/MT2-mediated SIRT1/Nrf2 signaling pathway. Our results show that platinum chemotherapeutic drugs can lead to testicular injury, decreased testosterone levels, decreased sperm concentrations, and increased oxidative stress. The contents of melatonin and melatonin synthase AANAT and ASMT also decreased. Melatonin pretreatment can alleviate cisplatin-induced oxidative damage in mice. Melatonin regulates the SIRT1/Nrf2 pathway through the melatonin receptors (MT1/MT2) and plays an important role in protecting against the cisplatin-induced apoptosis of mouse Leydig cells (Figure 12).

## Data Availability

The data presented in this study are available in this manuscript.

## Supplementary Materials

The following supporting information can be downloaded at: https://www.mdpi.com/article/10.3390/cells11101690/s1, Figure S1: Localization and expression of MT1 and MT2 in testicular tissue. Figure S2: Localization and expression of MT1 and MT2 in mice during different recovery periods after cisplatin/non-cisplatin treatment.

## Abbreviations

CAT, catalase; CP, cisplatin; DEPs, differently expressed proteins; FSH, follicle-stimulating hormone; GSH, reduced glutathione; GSH-Px, glutathione peroxidase; HO-1, heme oxygenase 1; LH, Luteinizing hormone; Mel; melatonin; MDA, malondialdehyde; MT1, melatonin receptor 1; MT2, melatonin receptor 2; NQO1, NAD(P)H quinone oxidoreductase-1; Nrf2, nuclear factor erythroid 2-related factor 2; ROS, reactive oxygen species; SIRT1, silencing information regulator 1; SOD, superoxide dismutase; SOD1, Cu/Zn superoxide dismutase; SOD2, manganese superoxide dismutase; T, testosterone.
