# Supplementary material for: Activation of MT1/MT2 to Protect Testes and Leydig Cells against Cisplatin-Induced Oxidative Stress through the SIRT1/Nrf2 Signaling Pathway
Source: Cells. 2022 May 19;11(10):1690. doi: 10.3390/cells11101690 (PMC9139217; doi:10.3390/cells11101690)
Supplement: Supplementary file 1 [file cells-11-01690-s001.zip › Supplementary Figure-0520doc.pdf]

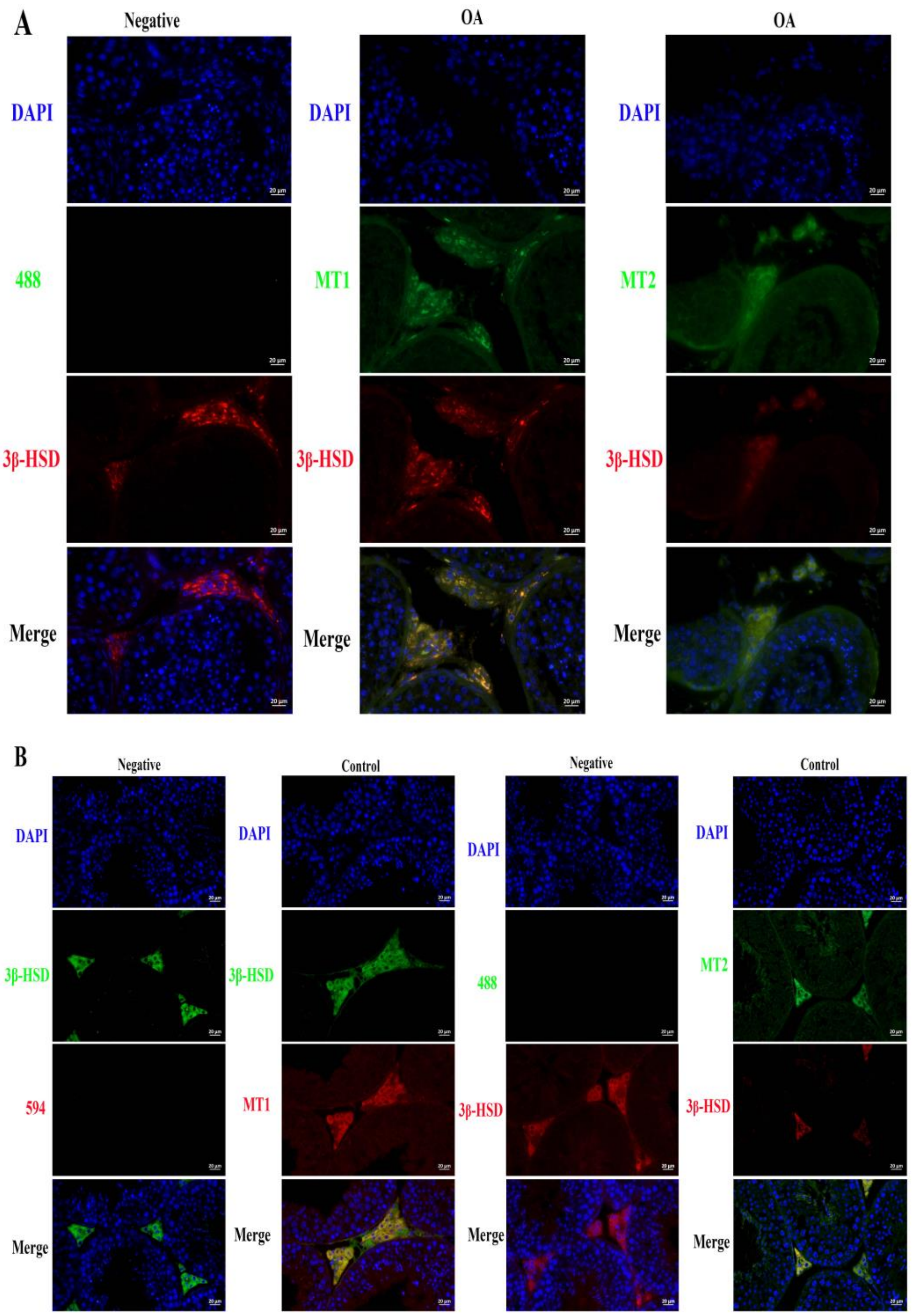

Supplementary Figure S1. Localization and expression of MT1 and MT2 in testicular tissue. (**A**) Immunofluorescent pictures of MT1 and MT2 in human testicular tissue are representative. 3β-HSD was tagged with Alexa Fluor 594, while MT1/MT2 were tagged with Alexa Fluor 488. The nucleus was labeled with DAPI (scale bar = 20 μm). (**B**) Immunofluorescent pictures of MT1/MT2 in mouse testicular tissue. 3β-HSD was tagged with Alexa Fluor 488 or Alexa Fluor 594, while MT1/MT2 were tagged with Alexa Fluor 594 or Alexa Fluor 488. The nucleus was labeled with DAPI (scale bar = 20 μm).

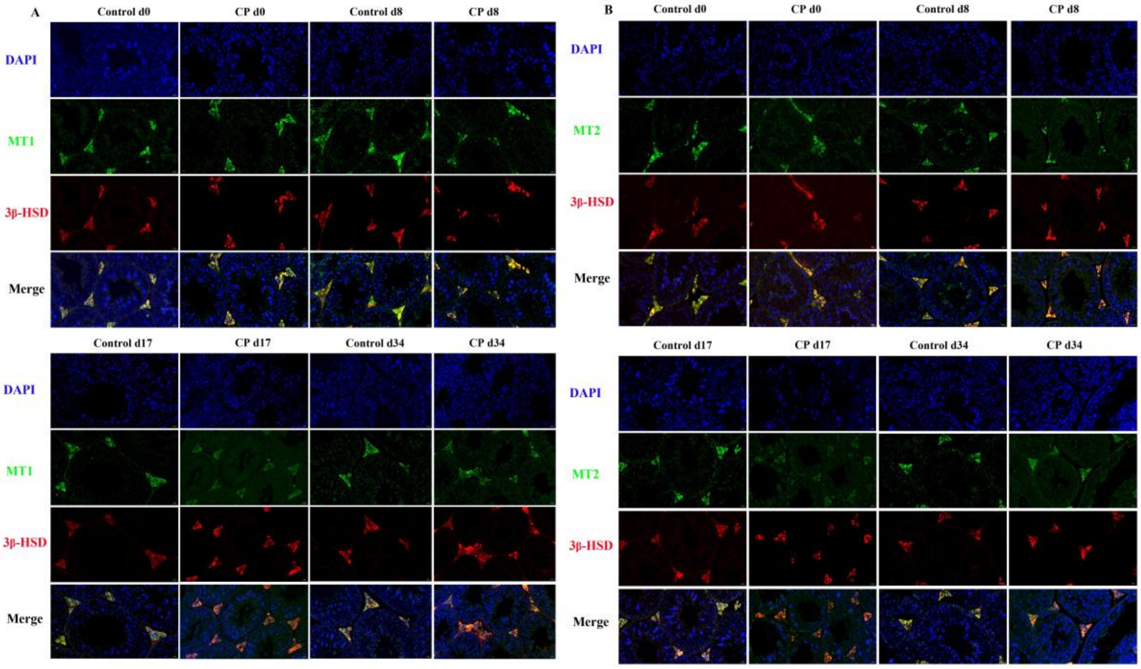

Supplementary Figure S2. Localization and expression of MT1 and MT2 in mice during different recovery periods after cisplatin/non-cisplatin treatment. (**A**) Immunofluorescent pictures of MT1 in mouse testicular tissue are representative. 3β-HSD was tagged with red fluorescence, while MT1 was tagged with green fluorescence. The nucleus was labeled with DAPI (scale bar = 20 μm). (**B**) Immunofluorescent pictures of MT2 in mouse testicular tissue are representative. 3β-HSD was tagged with red fluorescence, while MT2 was tagged with green fluorescence. The nucleus was labeled with DAPI (scale bar = 20 μm).
